# Do case‐only designs yield consistent results across design and different databases? A case study of hip fractures and benzodiazepines†

**DOI:** 10.1002/pds.3822

**Published:** 2015-06-26

**Authors:** Gema Requena, John Logie, Elisa Martin, Nada Boudiaf, Rocío González González, Consuelo Huerta, Arturo Alvarez, David Webb, Andrew Bate, Luis A. García Rodríguez, Robert Reynolds, Raymond Schlienger, Helga Gardarsdottir, Mark de Groot, Olaf H. Klungel, Fancisco de Abajo, Ian J. Douglas

**Affiliations:** ^1^ Pharmacology Unit, Department of Biomedical Sciences, School of MedicineUniversity of AlcaláMadridSpain; ^2^ Worldwide EpidemiologyGlaxoSmithKlineResearch and DevelopmentUxbridgeMiddlesexUK; ^3^ BIFAP Research UnitSpanish Agency of Medicines and Medical DevicesMadridSpain; ^4^ EpidemiologyPfizer LtdTadworthUK; ^5^ Spanish Center for Pharmacoepidemiological Research (CEIFE)MadridSpain; ^6^ EpidemiologyPfizer Research and DevelopmentNew YorkUSA; ^7^ Global Clinical EpidemiologyNovartis Pharma AGBaselSwitzerland; ^8^ Division of Pharmacoepidemiology and Clinical PharmacologyUtrecht UniversityUtrechtthe Netherlands; ^9^ Clinical Pharmacology UnitUniversity Hospital Príncipe de AsturiasMadridSpain; ^10^ London School of Hygiene and Tropical Medicine (LSHTM)LondonUK

**Keywords:** case crossover (CXO), self‐controlled case series (SCCS), benzodiazepines, hip fractures, electronic healthcare records databases (DBs), pharmacoepidemiology

## Abstract

**Background:**

The case‐crossover (CXO) and self‐controlled case series (SCCS) designs are increasingly used in pharmacoepidemiology. In both, relative risk estimates are obtained within persons, implicitly controlling for time‐fixed confounding variables.

**Objectives:**

To examine the consistency of relative risk estimates of hip/femur fractures (HFF) associated with the use of benzodiazepines (BZD) across case‐only designs in two databases (DBs), when a common protocol was applied.

**Methods:**

CXO and SCCS studies were conducted in BIFAP (Spain) and CPRD (UK). Exposure to BZD was divided into non‐use, current, recent and past use. For CXO, odds ratios (OR; 95%CI) of current use versus non‐use/past were estimated using conditional logistic regression adjusted for co‐medications (AOR). For the SCCS, conditional Poisson regression was used to estimate incidence rate ratios (IRR; 95%CI) of current use versus non/past‐use, adjusted for age. To investigate possible event‐exposure dependence the relative risk in the 30 days prior to first BZD exposure was also evaluated.

**Results:**

In the CXO current use of BZD was associated with an increased risk of HFF in both DBs, AOR_BIFAP_ = 1.47 (1.29–1.67) and AOR_CPRD_ = 1.55 (1.41–1.70). In the SCCS, IRRs for current exposure was 0.79 (0.72–0.86) in BIFAP and 1.21 (1.13–1.30) in CPRD. However, when we considered separately the 30‐day pre‐exposure period, the IRR for current period was 1.43 (1.31–1.57) in BIFAP and 1.37 (1.27–1.47) in CPRD.

**Conclusions:**

CXO designs yielded consistent results across DBs, while initial SCCS analyses did not. Accounting for event‐exposure dependence, estimates derived from SCCS were more consistent across DBs and designs. © 2015 The Authors. Pharmacoepidemiology and Drug Safety published by John Wiley & Sons Ltd.

## Introduction

Case‐only designs overcome some key confounding issues such as lack of information on potential confounders, and difficulties in selecting appropriate controls in numerous settings.^1^ One common characteristic of those designs is that comparisons are within‐person not between‐persons, thereby controlling implicitly for all intrinsic factors, both measured and unmeasured, that remain constant over the study period. For these reasons they are increasingly being used in pharmacoepidemiology.^2^

The case‐crossover (CXO) method was developed by Maclure (1991), to investigate the risk of transient and immediate acute events.^3^ The particularity is that ‘controls’ come from the person‐time of the case. It uses the difference in exposure rates just before the event (the ‘case moment’) with those at other times (‘controls moments’) to estimate an odds ratio (OR) of the outcome associated with exposure. It depends on strong assumptions, being suitable for transient exposures with short term effects.^4^ Hence, the intermittency of drug use and the length of the exposure time window may have an impact on the estimates obtained.^2^ Also, as a conditional logistic regression model is employed with more than one control moment, distribution of exposures must be exchangeable between those periods to emulate a case–control design where the order of controls is irrelevant.^5^

The self‐controlled case series (SCCS) method was developed by Farrington (1995) to study the association between vaccination and adverse events.^6^ The SCCS follows the cohort design approach; it is derived from a Poisson distribution model by conditioning on an individual's total number of events and their exposure history.^7^

The SCCS is based on several assumptions; one is that the occurrence of the event of interest does not influence the chance of subsequent exposure. However, in situations where the event could temporarily increase or decrease the likelihood of exposure, a valid approach is to separately categorise a short period of time before exposure, thereby removing this time from the reference category (baseline or period of no exposure) to avoid a biassed event rate in that period.

Because the designs share some features but follow a different approach, we assessed whether they reached similar results across electronic healthcare records databases (DBs) from the United Kingdom (UK) and Spain following the same protocol and methodology. As a case study we used the well‐established association^8^, ^9^ of benzodiazepines and related drugs (BZD) with hip/femur fractures (HFF). BZD are often used intermittently, and with these case‐only designs some confounding such as frailty could be addressed.

The present research was undertaken within the frame of the Pharmacoepidemiological Research on Outcomes of Therapeutics by a European Consortium (IMI‐PROTECT) project (http://www.imi‐protect.eu/).

## Patients and Methods

The study was performed in two primary care DBs: The UK Clinical Practice Research Datalink (CPRD GOLD),^10^ and ‘Base de datos para la Investigación Farmacoepidemiológica en Atención Primaria’ (BIFAP) from Spain. These DBs have been described in detail elsewhere.^11^ The protocol was registered in The European Network of Centres for Pharmacoepidemiolgy and Pharmacovigilance, ENCePP.^12^ A blinding procedure was maintained until results were made available to the coordinating centre at Utrecht University, the Netherlands.

### Study population

The study period was considered from the 1 January 2001 until 31 December 2009. All data were used when the practices were considered ‘Up to (research) Standard’ (a marker of data quality). Patients who had at least one year of registration with the general practitioner (GP), were ≥18 years old, and were 12 months free of HFF were included in the study population. All patients were required to have a recorded diagnosis of HFF during the study period, i.e. they were all ‘cases’. For the SCCS, patients were required to have 6 months free of BZD prescriptions before entering the study to restrict the population to new users. This criterion was not applied for the CXO to ensure that all case or control moments had the opportunity to be exposed to BZD. Patients could enter at any time they fulfilled the criteria above. The start date was the date patients met the cited criteria.

### Case definition

HFF was searched in the BIFAP database using the International Classification of Primary Care: ICPC‐2, code L75 and in CPRD using READ codes (Table S1 online). In BIFAP, cases were identified through free‐text (in addition to codes). For that reason, a review of all cases was carried out for validation. As a result, similar to the companion Cohort/NCC paper,^13^ 30% of cases were excluded (of them, about 15% because of high‐energy trauma, 60% because of other fractures (i.e. pelvis), and the remaining patients did not have a clear date of the event). Such a revision was not feasible in the CPRD, but previous validation confirmed 91% of recorded hip fractures in the CPRD.^14^ In patients with a history of past HFF, a minimum of 12 months must have elapsed between the current episode and any previous fracture to ensure these represented separate events.

### Exposure definition

BZD was the exposure of interest, comprising all those classified as anxiolytics, hypnotics and related drugs in the Anatomical Therapeutic Chemical (ATC) classification.^15^ (Supplementary Table S2 online). Related drugs (Z‐drugs and clomethiazole) were included in this research because their therapeutic actions are similar to benzodiazepines.^16^

Duration of each prescription was estimated based on the prescribed amount and daily dose. The expected duration of use was calculated following the methods of Gardarsdottir *et al*.^17^ When a gap of more than 30 days occurred between the theoretical end date of a prescription and the date of the subsequent prescription, exposure was considered to be discontinuous, and a new treatment episode was considered.

**Figure 1:**
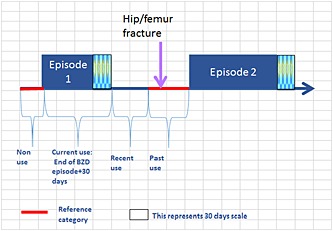
Structure of exposure definition. The follow‐up continued beyond the date of hip/femur fracture only for the SCCS

The person‐time of each patient was divided according to their exposure into periods of current, recent, past and non‐use. Thus, current use was the period from the start of a BZD prescription until 30 days after the estimated end date of the supply; recent use was the period up to 60 days after current use; past use was the period after recent use until the patient became exposed again or the end of follow‐up; non‐use was the period between the start date and the first BZD prescription within the study period. Combined non‐use and past use was considered the reference category or baseline (Figure 1). For the SCCS, current use was further divided into five risk time windows: 1–30, 31–60, 61–182, 183–365 and >365 days. BZD are thought to increase the risk of fractures during the early stages of treatment,^18^, ^19^ and this was taken into account when defining exposure time windows.

For the CXO, each case serves as its own control, and up to four control moments were defined at 91, 182, 273 and 365 days prior to the HFF (case moment). This method assumes that the baseline risk for an exposure is constant, and this assumption was tested using up to four control periods per case, improving the precision of the effect size, and the efficiency by using the whole year prior to the event.^20^

For each patient, exposure at the case moment was compared to exposure at control moments. In addition for this design, the current use period was further categorised as single use of anxiolytics, single use of hypnotics and use of both.

### Potential confounders

These studies are part of a common protocol where four analytical study designs were performed to investigate the same study question (http://www.encepp.eu/encepp/viewResource.htm?id=6179).

In the CXO, all medications mentioned in the protocol (see Table S3 online) were considered as potential confounders. Indicator terms for medication use were added to the model denoting the absence or presence of prescriptions of each separate type of medication listed in Table S3, within the 91 days prior to the case or control moments.

In the SCCS, age was considered as the most important potential confounder, given its strong association with fracture risk^21^ and given that many relevant unmeasured factors are likely to be age‐related (e.g. frailty or increase in severity of underlying diseases) as well as related to BZD pattern of use.

### Analysis

Analyses in BIFAP were performed using Stata®‐11; in CPRD analyses were performed using SAS v9.2 for the CXO and Stata®‐10 for the SCCS.

In the CXO, conditional logistic regression was used to estimate the relative risk in terms of ORs with corresponding 95% confidence intervals (CI).

In the SCCS, conditional Poisson regression was used to estimate the relative risk in terms of incidence rate ratios (IRRs) with corresponding 95% CI.^22^

To examine potential event‐exposure dependence, a pre‐exposure time risk window was created in the SCCS with a length of 30 days, allowing us to examine whether an incident HFF has a short‐term impact on the likelihood of being prescribed a BZD. IRR were estimated excluding this pre‐exposure time of 30 days from the reference category in a sensitivity analysis.

## Results

#### Case crossover

##### Characteristics of study populations (BIFAP and CPRD)

In BIFAP, 5412 cases were included, with a similar mean age of 78 (±13) years old. From these, 85% contributed four control moments (Table 1). In CPRD, a total of 12 853 cases of HFF were included as the study population, with a mean age (±SD) of 79 (±13) years old. From these, 88% were also registered in the DB during the four control moments. Distribution by sex was similar in both DBs, 78% females and 22% males. The characteristics of patients are described in Table S4 online.

**Table 1:** Number of cases and its control moments participating in case‐crossover study in BIFAP and CPRD

	Number of cases with M controls (N%)		Number of cases with at least M controls (N%)	
Control moments (M)	CPRD	BIFAP	CPRD	BIFAP
**1**	530 (4.1)	267 (4.7)	12 853 (100)	5412 (100)
**2**	474 (3.7)	272 (4.8)	12 323 (95.9)	5145 (95.1)
**3**	492 (3.8)	274 (4.8)	11 849 (92.2)	4873 (90.0)
**4**	11 357 (88.4)	4599 (80.6)	11 357 (88.4)	4599 (85.0)

##### Effect of BZD (BIFAP and CPRD)

**Figure 2:**
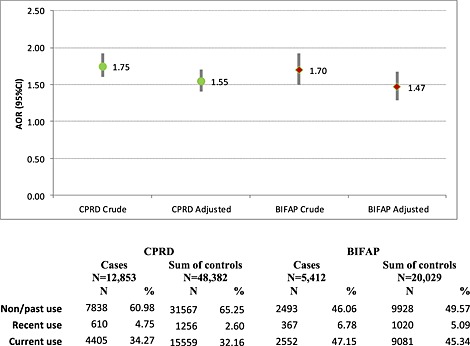
Crude and co‐medication adjusted risk of hip/femur fracture associated to current use of BZD. Case‐crossover study

Crude ORs (95%CI) and adjusted OR (AORs) (95%CI) for current use of BZD, compared to past/non‐use, were similar between DBs: OR = 1.70 (1.50–1.92), AOR = 1.47 (1.29–1.67) in BIFAP and OR = 1.75 (1.60–1.92), AOR = 1.55 (1.41–1.70) in CPRD (Figure 2).

##### Effect of BZD treatment duration in CPRD

**Figure 3:**
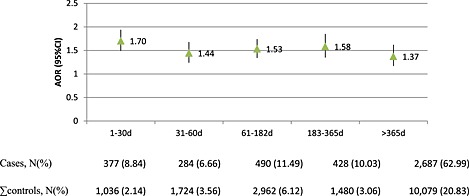
Co‐medication adjusted risk of hip/femur fracture associated to duration of current use of BZD in CPRD. Case‐crossover study

In CPRD, although the highest relative risk was observed within the first 30 days of treatment AOR (95%CI): 1.70 (1.49–1.94), a model accounting for duration class did not provide a significantly better fit to the data than one considering presence/absence of BZD exposure alone (chi‐square for comparison of −2log L scores = 6.82, DF = 4, p = 0.15) (Figure 3).

#### Self‐controlled case series

##### Characteristics of study populations (BIFAP and CPRD)

In CPRD, a total of 8333 cases were included as the study population, and a total of 4450 cases were included in BIFAP. In both populations the age and gender distribution was similar; 77% and 74% were females in BIFAP and CPRD respectively. The mean age at first exposure to BZD was about 76 years old in both DBs. The percentage of HFF occurring during the exposure to BZD (current use) was higher in BIFAP (35%) than in CPRD (22%), and the median duration of the observation period was shorter in BIFAP (5.4 years) than in CPRD (7.0 years). Of 8333 patients exposed to BZD in CPRD, 4790 had only a single continuous period of BZD exposure compared to 3543 patients (42.52%) who had intermittent BZD use. In the same way, of 4450 patients exposed to BZD in BIFAP, 1782 had only a single continuous period of BZD exposure compared to 2668 (59.96%) who had intermittent BZD use.

##### Effect of BZD and related drugs

Adjusted IRR of HFF associated with current use was 1.21 (1.13–1.30) in CPRD and 0.79 (0.72–0.86) in BIFAP in analyses ignoring the potential for event‐exposure dependence. In CPRD, an apparent decreasing trend of risk with duration of treatment was observed, ranging from 1.42 (1.27–1.59) in the first 30 days of use to 0.89 (0.79–1.02) with >365 days of use. In BIFAP, no increased risk was observed in any time window category (Table 2).

**Table 2:** Risk of hip/femur fracture associated with current use of BZD from SCCS studies in BIFAP and CPRD without adjustment for event‐exposure dependence

	BIFAP (N = 4450)								CPRD (N = 8333)							
			Model crude			Model adjusted by age					Model crude			Model adjusted by age		
	Cases	Follow‐up days	IRR	IC (95%)		IRR	IC (95%)		Cases	Follow‐up days	IRR	IC (95%)		IRR	IC (95%)	
Past/non‐use	2615	5 169 915	1.00	—	—	1.00	—	—	6060	15 769 427	1.00	—	—	1.00	—	—
Recent use	292	476 872	1.14	1.00	1.29	0.97	0.85	1.10	439	792 974	1.42	1.28	1.57	1.26	1.14	1.39
Current use	1543	2 945 532	1.02	0.94	1.11	0.79	0.72	0.86	1834	3 274 091	1.55	1.45	1.66	1.21	1.13	1.30
1–30 days	213	409 985	0.92	0.80	1.07	0.79	0.68	0.92	342	510 624	1.59	1.42	1.78	1.42	1.27	1.59
31–60 days	201	362 943	0.99	0.85	1.15	0.85	0.73	0.99	253	425 445	1.44	1.27	1.64	1.27	1.11	1.44
61–182 days	314	614 880	0.93	0.82	1.06	0.75	0.66	0.86	383	647 956	1.48	1.33	1.66	1.19	1.06	1.33
183–365 days	246	437 601	1.11	0.95	1.29	0.83	0.71	0.96	300	498 354	1.66	1.46	1.89	1.20	1.05	1.37
>365 days	569	1 120 123	1.28	1.12	1.47	0.73	0.63	0.84	556	1 191 712	1.62	1.43	1.82	0.89	0.79	1.02

##### Sensitivity analysis of event‐exposure dependence

When a 30‐day pre‐exposure window was removed from the reference period, an increased risk was observed across all exposure period time windows. The highest risk was exhibited by the pre‐exposure time window, with 2.52 (95%CI: 2.30–2.76) and 6.47 (95%CI: 5.91–7.09) adjusted for age, in CPRD and BIFAP, respectively. Excluding this pre‐exposure time from the reference category we observed in BIFAP a marked change in the magnitude of the estimates, over all exposure time windows, reaching an IRR of 1.43 (1.31–1.57) when current use was aggregated in just one category. Such increment was less for CPRD varying from 1.21 (1.13–1.30) to 1.37 (1.27–1.47) in the current use category (Table 3).

**Table 3:** Risk of hip/femur fracture associated with current use of BZD from SCCS studies in BIFAP and CPRD including adjustment for event‐exposure dependence

	BIFAP (N = 4450)								CPRD (N = 8333)							
			Model crude			Model adjusted by age					Model crude			Model adjusted by age		
	Cases	Follow‐up days	IRR	IC (95%)		IRR	IC (95%)		Cases	Follow‐up days	IRR	IC (95%)		IRR	IC (95%)	
Past/non‐use	1898	4 941 912	1.00	—	—	1.00	—	—	5549	15 344 912	1	—	—	1	—	—
Recent use	172	361 218	1.37	1.16	1.60	1.21	1.03	1.42	401	712 289	1.58	1.43	1.76	1.41	1.27	1.57
Current use	1543	2 945 532	1.77	1.62	1.93	1.43	1.31	1.57	1834	3 274 091	1.75	1.63	1.87	1.37	1.27	1.47
1–30 days	213	409 985	1.58	1.36	1.83	1.40	1.21	1.62	342	510 624	1.79	1.60	2.00	1.59	1.42	1.78
31–60 days	201	362 943	1.69	1.45	1.97	1.49	1.28	1.74	253	425 445	1.62	1.43	1.85	1.42	1.25	1.62
61–182 days	314	614 880	1.63	1.43	1.86	1.37	1.20	1.57	383	647 956	1.68	1.50	1.89	1.35	1.20	1.51
183–365 days	246	437 601	1.95	1.68	2.27	1.53	1.32	1.79	300	498 354	1.88	1.65	2.14	1.36	1.19	1.55
>365 days	569	1 120 123	2.27	1.97	2.60	1.42	1.22	1.65	556	1 191 712	1.83	1.62	2.06	1.02	0.90	1.16
Pre‐exposure period	837	343 657	7.17	6.55	7.84	6.47	5.91	7.09	549	505,200	2.83	2.59	3.11	2.52	2.30	2.76

## Discussion

Under PROTECT's framework of Pharmacoepidemiology studies, four analytical designs were performed in different databases focusing on the methodological aspects of the studies rather than the clinical consequences of the association under investigation. Two case‐only designs, studying the association of BZD with HFF, are presented here. The results of the cohort and nested case–control (NCC) studies for the same association are presented elsewhere.^13^

##### CXO study

Crude and AORs were similar between databases. Other CXO studies showed similar associations. Neutel *et al*.,^23^ for example, found a crude OR = 1.7, 95%CI: 1.0–2.9, for exposure to BZD and Berry *et al*.^24^ found an AOR = 1.66, 95%CI: 1.45–1.90 associated with the use of non‐BZD hypnotics.

Concerning the *effect of BZD subgroup*, in both DBs the highest risk was observed in patients taking both anxiolytics and hypnotics, similar to the results seen with other study designs evaluated in PROTECT.^13^ As falls and fractures are dose‐related adverse effects,^25^ the use of several drugs could be seen as equivalent to the use of a higher dose. Alternatively, this could partly be related to the higher severity of the underlying conditions of these patients.

Regarding the *effect of BZD treatment duration* in CPRD, although the highest relative risk was observed within the first 30 days of treatment AOR (95%CI): 1.70 (1.49–1.94), a duration effect was not supported by formal test (p = 0.15). There is a lack of published articles exploring this short‐term effect of BZD and related drugs with this design. This method assumes immediate and transient effect as well as intermittent exposures and the power to detect the effect of continuous treatment may be limited. Possible explanations for increasing or decreasing the risk with duration of treatment have been discussed previously.^13^

In BIFAP, it was not possible to examine duration of use in this design because data available before the study period were insufficient for assessing all duration categories.

##### SCCS study

An increased risk of HFF with the use of BZD was observed in CPRD but not in any exposure category in initial analyses after adjusting for age in BIFAP (Table 2).

However, within these analyses, we found evidence of a strong but temporary dependence of event and exposure implying that some patients who sustained a HFF were prescribed a BZD shortly after the event. This dependence violates one of the key assumptions of this design. Separating a 30‐day period from the reference category, the results in the SCCS were similar to the CXO in both BIFAP and CPRD, again suggesting an increased fracture risk associated with exposure to BZD in all current use windows.

Gibson *et al*.^22^ also used a pre‐exposure time to assess the risk of motor vehicle crashes with BZD, and found elevated risks (IRR = 1.94, 99% CI: 1.62, 2. 32). A similar situation was observed by Lai C *et al*.^14^ studying the risk of HFF associated with alpha blockers using a SCCS design.

##### Comparison across all designs

Both traditional (cohort and NCC) and case‐only designs suggested an increased risk of HFF associated with current use of BZD. However, designs differed in the magnitude of risk with traditional designs showing slightly lower relative risks (RR) than case‐only designs. Differences observed between designs might be because of the fact that chronic users of BZD with no unexposed observation time are excluded from the estimated RR in the case‐only designs, but can contribute in cohort and NCC analyses. Results must be interpreted in the light of these differences and in some instances, case‐only results may be more accurately generalised to intermittent users. If chronic users of BZD had a lower risk than short term users, explained by a better adaptation of regular users to those drugs or by the consequence of ‘healthy user effect’,^26^ traditional designs would be expected to yield lower RR estimates than case‐only designs. Conversely, traditional designs estimate between‐person RR, while case‐only estimate within‐person RR,^27^, ^28^ which may not be necessarily of the same magnitude because of unmeasured factors difficult to adjust for such as severity of underlying diseases, or frailty. Such factors may increase the risk of fall and fractures and may make physicians reluctant to prescribe BZD (e.g. confounding by contraindication). This confounding would lead to an underestimate of the relative risk of HFF associated with BZD use. In case‐only designs, time invariant confounding factors are implicitly controlled for by design, although a confounding by transient changes in other factors cannot be excluded.

The fact that the cohort/NCC analyses were restricted to BZD users with a reference category of past‐use, and therefore, all patients were exposed at least once to the drug of interest, made the comparison between cohort members more similar than if an external cohort of non‐users had been used. In contrast, a reference category of non‐use was appropriate for the case‐only analyses, as differences between persons are removed by design.

Results obtained with both case‐only designs were similar, although the precision of the estimates was higher in the SCCS than in the CXO which may be considered as potential advantage of the former.

The experience of comparing different designs using the same source population is limited. The study of Madigan *et al*.,^29^ part of the Observational Medical Outcomes Partnership (OMOP) project, studied this drug‐event pair employing a SCCS and a cohort design within the same data source, and compared the results across ten DBs. Seven out of ten DBs found no increased risk of HFF associated with BZD use, when a SCCS was employed. However, this study was not specific to BZD‐HFF, and explored 53 drug‐event pairs under a surveillance perspective rather than specifically addressing this pair in a formal hypothesis testing study with a pair specific protocol.

There are some publications comparing designs yielding different results, although studying different associations.^30^, ^31^ In most articles, relative risk estimates with case‐only designs were lower than those obtained with cohort or NCC designs, with authors generally concluding that the higher estimates obtained using these designs may be because of between‐person confounding.

##### Strengths and limitations

We only had access to prescribing data, rather than the precise dates on which medication was actually taken. It is therefore possible that exposure periods are misclassified to some extent, with both exposed and unexposed periods affected to some degree. The effect of this would tend to bias results towards the null, and so it is possible that we have underestimated any real effect of treatment with BZD. A major strength of this research is the use of a common protocol allowing the use of harmonised methods and definitions across DBs, aiding the direct comparison of results.

In general, case‐only designs are limited by their underlying assumptions. In this study, the assumption of independence between event and exposure in the SCCS design was not met but was subsequently corrected for by the use of a pre‐exposure risk period.^22^

In our case‐only two DBs have been employed with just one drug‐event association, so results might not be extrapolated to other settings and certainly not be generalised to other drug‐event pairs.

## Conclusions

CXO designs yielded consistent results across DBs. Once we accounted for the event‐exposure dependence, estimates derived from SCCS were also consistent across DBs and across designs. Case‐only designs may offer better control for time invariant confounding factors than traditional designs and are a useful choice when intrinsic factors may represent relevant confounding, and when the effects of transient exposures are to be measured. Care is needed to ensure the underlying assumptions of these designs are met and to interpret the results obtained as they may not always generalise to patients receiving continuous treatment with the medication being assessed.

These studies together with the cohort and case–control analyses have shown that performing multi‐site studies using a common protocol provides useful comparisons across countries and across designs, contributing to a better understanding of potential differences between pharmacoepidemiological studies used to assess drug safety.

## Conflict of Interest

OK and MdG have received unrestricted funding for pharmacoepidemiological research from the Dutch private‐public funded Top Institute Pharma.

JL, DW and NB are employees and stockholders of GlaxoSmithKline.

RS is a Novartis employee and owns Novartis shares.

AB and RR are employees and stockholders of Pfizer, Inc.

IJD consults for and holds stock in GSK, and consults for Gilead.

Key Points

- Case‐only designs may offer better control for time invariant confounding factors than traditional designs and may be a useful choice when intrinsic factors may represent relevant confounding.
- Care is needed to ensure that the underlying assumptions of these designs are met and to interpret the results obtained as they may not always generalise to patients receiving continuous treatment with the medication being assessed.
- In the SCCS design, it is important to explore the potential event‐exposure dependence as even temporary effects can have a large impact on results.
- This research has shown that performing multi‐site studies, using a common protocol provides useful comparisons across countries and across designs.

## Ethics Statement

Study protocols were approved by institutional review boards responsible for each individual database.

## Author Contributions

All authors contributed to the study conception and design. The corresponding authors responsible per DB performed data extraction and raw data analysis. GR, JL and IJD wrote the first draft, and all authors contributed with critical comments to the final version.

## Supporting information



Supporting info item
