# Supplementary material for: Do case‐only designs yield consistent results across design and different databases? A case study of hip fractures and benzodiazepines
Source: Pharmacoepidemiol Drug Saf. 2015 Jun 26;25(Suppl Suppl 1):79–87. doi: 10.1002/pds.3822 (PMC4949640; doi:10.1002/pds.3822)
Supplement: Supplementary file 1 — Supporting info item [file PDS-25-79-s001.pdf]

Table S1 online- ICPC-2 and READ Codes for hip/femur fracture (BIFAP and CPRD databases)

CODES	HIP/FEMUR FRACTURES
**ICPC-2**	
L75	Fracture: femur
**READ**	**HIP FRACTURES**
7K1L400	CLOSED REDUCTION OF FRACTURE OF HIP
S30..00	FRACTURE OF NECK OF FEMUR
S30..11	HIP FRACTURE
S300.00	CLOSED FRACTURE PROXIMAL FEMUR, TRANSCERVICAL
S300000	Cls # prox femur, intracapsular section, unspecified
S300100	CLOSED FRACTURE PROXIMAL FEMUR, TRANSEPIPHYSEAL
S300200	CLOSED FRACTURE PROXIMAL FEMUR, MIDCERVICAL SECTION
S300300	CLOSED FRACTURE PROXIMAL FEMUR, BASICERVICAL
S300311	CLOSED FRACTURE, BASE OF NECK OF FEMUR
S300400	CLOSED FRACTURE HEAD OF FEMUR
S300500	Cls # prox femur, subcapital, Garden grade unspec.
S300600	CLOSED FRACTURE PROXIMAL FEMUR, SUBCAPITAL, GARDEN GRADE I
S300700	CLOSED FRACTURE PROXIMAL FEMUR, SUBCAPITAL, GARDEN GRADE II
S300800	CLOSED FRACTURE PROXIMAL FEMUR, SUBCAPITAL, GARDEN GRADE III
S300900	CLOSED FRACTURE PROXIMAL FEMUR, SUBCAPITAL, GARDEN GRADE IV
S300A00	CLOSED FRACTURE OF FEMUR, UPPER EPIPHYSIS
S300y00	CLOSED FRACTURE PROXIMAL FEMUR, OTHER TRANSCERVICAL
S300y11	CLOSED FRACTURE OF FEMUR, SUBCAPITAL
S300z00	CLOSED FRACTURE PROXIMAL FEMUR, TRANSCERVICAL, NOS
S301.00	OPEN FRACTURE PROXIMAL FEMUR, TRANSCERVICAL
S301000	Opn # proximal femur, intracapsular section, unspecified
S301100	OPEN FRACTURE PROXIMAL FEMUR, TRANSEPIPHYSEAL
S301200	OPEN FRACTURE PROXIMAL FEMUR, MIDCERVICAL SECTION
S301300	OPEN FRACTURE PROXIMAL FEMUR, BASICERVICAL
S301311	OPEN FRACTURE BASE OF NECK OF FEMUR
S301400	OPEN FRACTURE HEAD, FEMUR
S301500	OPEN FRACTURE PROXIMAL FEMUR,SUBCAPITAL, GARDEN GRADE UNSPEC
S301600	OPEN FRACTURE PROXIMAL FEMUR,SUBCAPITAL, GARDEN GRADE I
S301700	OPEN FRACTURE PROXIMAL FEMUR,SUBCAPITAL, GARDEN GRADE II
S301800	OPEN FRACTURE PROXIMAL FEMUR,SUBCAPITAL, GARDEN GRADE III
S301900	OPEN FRACTURE PROXIMAL FEMUR,SUBCAPITAL, GARDEN GRADE IV
S301A00	OPEN FRACTURE OF FEMUR, UPPER EPIPHYSIS
S301y00	OPEN FRACTURE PROXIMAL FEMUR, OTHER TRANSCERVICAL
S301y11	OPEN FRACTURE OF FEMUR, SUBCAPITAL
S301z00	OPEN FRACTURE PROXIMAL FEMUR, TRANSCERVICAL, NOS
S302.00	CLOSED FRACTURE OF PROXIMAL FEMUR, PERTROCHANTERIC
S302000	Cls # proximal femur, trochanteric section, unspecified
S302011	CLOSED FRACTURE OF FEMUR, GREATER TROCHANTER
S302012	CLOSED FRACTURE OF FEMUR, LESSER TROCHANTER
S302100	CLOSED FRACTURE PROXIMAL FEMUR, INTERTROCHANTERIC, TWO PART
S302200	CLOSED FRACTURE PROXIMAL FEMUR, SUBTROCHANTERIC
S302300	Cls # proximal femur, intertrochanteric, comminuted
S302400	CLOSED FRACTURE OF FEMUR, INTERTROCHANTERIC

S302z00	Cls # of proximal femur, pertrochanteric section, NOS
S303.00	OPEN FRACTURE OF PROXIMAL FEMUR, PERTROCHANTERIC
S303011	OPEN FRACTURE OF FEMUR, GREATER TROCHANTER
S303012	OPEN FRACTURE OF FEMUR, LESSER TROCHANTER
S303100	OPEN FRACTURE PROXIMAL FEMUR, INTERTROCHANTERIC, TWO PART
S303200	OPEN FRACTURE PROXIMAL FEMUR, SUBTROCHANTERIC
S303300	OPEN FRACTURE PROXIMAL FEMUR, INTERTROCHANTERIC, COMMINUTED
S303400	OPEN FRACTURE OF FEMUR, INTERTROCHANTERIC
S303z00	OPEN FRACTURE OF PROXIMAL FEMUR, PERTROCHANTERIC, NOS
S304.00	PERTROCHANTERIC FRACTURE
S305.00	SUBTROCHANTERIC FRACTURE
S30w.00	CLOSED FRACTURE OF UNSPECIFIED PROXIMAL FEMUR
S30x.00	OPEN FRACTURE OF UNSPECIFIED PROXIMAL FEMUR
S30y.00	CLOSED FRACTURE OF NECK OF FEMUR NOS
S30y.11	HIP FRACTURE NOS
S30z.00	OPEN FRACTURE OF NECK OF FEMUR NOS
S4E..00	FRACTURE-DISLOCATION OR SUBLUXATION HIP
S4E0.00	CLOSED FRACTURE-DISLOCATION, HIP JOINT
S4E1.00	OPEN FRACTURE-DISLOCATION, HIP JOINT
S4E2.00	CLOSED FRACTURE-SUBLUXATION, HIP JOINT
S4E3.00	OPEN FRACTURE-SUBLUXATION, HIP JOINT
**READ**	**FEMUR FRACTURES**
7K1G200	Primary open reduction+external fixation of femoral fracture
7K1L500	CLOSED REDUCTION OF FRACTURE OF FEMUR
K7805F	REDUCTION CLOSED FRACTURE FEMUR
K7815F	REDUCTION OPEN FRACTURE FEMUR
S31..00	OTHER FRACTURE OF FEMUR
S310.00	CLOSED FRACTURE OF FEMUR, SHAFT OR UNSPECIFIED PART
S310000	CLOSED FRACTURE OF FEMUR, UNSPECIFIED PART
S310100	CLOSED FRACTURE SHAFT OF FEMUR
S310011	Thigh fracture NOS
S310012	Upper leg fracture NOS
S310100	Closed fracture shaft of femur
S310z00	Closed fracture of shaft or unspecified part, NOS
S311.00	OPEN FRACTURE OF FEMUR, SHAFT OR UNSPECIFIED PART
S311000	OPEN FRACTURE OF FEMUR, UNSPECIFIED PART
S311100	OPEN FRACTURE SHAFT OF FEMUR
S311z00	OPEN FRACTURE OF FEMUR, SHAFT OR UNSPECIFIED PART, NOS
S312.00	CLOSED FRACTURE DISTAL FEMUR
S312.11	CLOSED FRACTURE OF FEMUR, DISTAL END
S312000	CLOSED FRACTURE OF DISTAL FEMUR, UNSPECIFIED
S312100	Closed fracture of femoral condyle, unspecified
S312200	CLOSED FRACTURE OF FEMUR, LOWER EPIPHYSIS
S312300	CLOSED FRACTURE DISTAL FEMUR, SUPRACONDYLAR
S312400	CLOSED FRACTURE DISTAL FEMUR, MEDIAL CONDYLE
S312500	CLOSED FRACTURE DISTAL FEMUR, LATERAL CONDYLE
S312600	CLOSED FRACTURE DISTAL FEMUR, BICONDYLAR (T-Y FRACTURE)
S312x00	CLOSED FRACTURE DISTAL FEMUR, COMMINUTED/INTRA-ARTICULAR
S312z00	CLOSED FRACTURE OF DISTAL FEMUR NOT OTHERWISE SPECIFIED
S313.00	OPEN FRACTURE DISTAL FEMUR
S313.11	OPEN FRACTURE OF FEMUR, DISTAL END
S313000	OPEN FRACTURE DISTAL FEMUR, UNSPECIFIED

S313100	OPEN FRACTURE OF FEMORAL CONDYLE, UNSPECIFIED
S313200	OPEN FRACTURE OF FEMUR, LOWER EPIPHYSIS
S313300	OPEN FRACTURE DISTAL FEMUR, SUPRACONDYLAR
S313400	OPEN FRACTURE DISTAL FEMUR, MEDIAL CONDYLE
S313500	OPEN FRACTURE DISTAL FEMUR, LATERAL CONDYLE
S313600	OPEN FRACTURE DISTAL FEMUR, BICONDYLAR (T-Y FRACTURE)
S313x00	OPEN FRACTURE DISTAL FEMUR, COMMINUTED/INTRA-ARTICULAR
S313z00	OPEN FRACTURE OF DISTAL FEMUR NOT OTHERWISE SPECIFIED
S314.00	FRACTURE OF SHAFT OF FEMUR
S315.00	FRACTURE OF LOWER END OF FEMUR
S31z.00	FRACTURE OF FEMUR, NOS
S3x2.00	MULTIPLE FRACTURES OF FEMUR
SC3D400	SEQUELAE OF FRACTURE OF FEMUR
Syu7200	[X]FRACTURES OF OTHER PARTS OF FEMUR

Table S2 online- List of benzodiazepines, DDD, and Half-life.

ATC code	Name	Defined Daily Dose DDD	Unit	Half-life*
N05B				
N05BA01	diazepam	10	mg	Long (>24)
N05BA02	chlordiazepoxide	30	mg	Long (>24)
N05BA03	medazepam	20	mg	Long (>24)
N05BA04	oxazepam	50	mg	Intermediate (8-24)
N05BA05	potassium clorazepate	20	mg	Long (>24)
N05BA06	lorazepam	2.5	mg	Intermediate (8-24)
N05BA07	adinazolam			Short (<8)
N05BA08	bromazepam	10	mg	Intermediate (8-24)
N05BA09	clobazam	20	mg	Intermediate (8-24)
N05BA10	ketazolam		mg	Intermediate (8-24)
N05BA11	prazepam	30	mg	Long (>24)
N05BA12	alprazolam	1	mg	Intermediate (8-24)
N05BA13	halazepam	0.1	g	Long (>24)
N05BA14	pinazepam		mg	Intermediate (8-24)
N05BA15	camazepam	30	mg	Intermediate (8-24)
N05BA16	nordazepam	15	mg	Long (24)
N05BA17	fludiazepam	0.75	mg	Long (>24)
N05BA19	etizolam		mg	Short (<8)
N05BA21	clotiazepam		mg	Short (<8)
N05CD				
N05CD01	flurazepam	30	mg	Long (>24)
N05CD02	nitrazepam	5	mg	Long(>24)
N05CD03	flunitrazepam	1	mg	Intermediate (8-24)
N05CD04	estazolam	3	mg	Intermediate (8-24)
N05CD05	triazolam	0.25	mg	Short(<8)
N05CD06	lormetazepam	1	mg	Intermediate(8-24)
N05CD07	temazepam	20	mg	Intermediate(8-24)
N05CD08	midazolam	15	mg	Short(<8)
N05CD09	brotizolam	0.25	mg	Short (<8)
N05CD10	quazepam	15	mg	Long(>24)
N05CD11	loprazolam	1	mg	Intermediate(8-24)
N05CF				
N05CF01	zopiclone*	7.5	mg	Short (<8)
N05CF02	zolpidem*	10	mg	Short (<8)
N05CF03	zaleplon	10	mg	Short (<8)
N05CM				
N05CM02	Clomethiazole	1.5	g	

* **Half life** definitions: Short (<8); Intermediate (8-24), Long (>24)

Table S3 online – List of medication codes included as potential confounders

ATC code	
H02AB	Glucocorticoids
M05BA01	etidronic acid
M05BA02	clodronic acid
M05BA03	pamidronic acid
M05BA04	alendronic acid
M05BA05	tiludronic acid
M05BA06	ibandronic acid
G03XC01	raloxifene
H05AA	Parathyroid hormones and analogues
M05BX03	Strontium ranelate
A11CC04	calcitriol
A11CC05	colecalciferol
	calcium+ colecalciferol
A11CC06	calcifediol
H05BA	Calcitonin preparations
N06AA	Non-selective monoamine reuptake inhibitors
N06AB	Selective serotonin reuptake inhibitors
N05A	Antipsychotics
N05AA	Phenothiazine with aliphatic side-chain
N05AB	Phenothiazines with piperazine structure
N05AC	Phenothiazines with piperidine structure
N05AD	Butyrophenone derivatives
N05AE	Indole derivatives
N05AF	Thioxanthene derivative
N05AG	Diphenylbutylpiperidine derivatives
N05AH	Diazepines, oxazepines, thiazepines and oxepines
N05AL	Benzamides
N05AN	Lithium
N05AX	Other antipsychotics
N04	Anti-Parkinson drugs
N04A	Anticholinergic agents
N04AA	Tertiary amines
N04AB	Ethers chemically close to antihistamine
N04AC	Ethers of tropine or tropine derivatives
N04B	Dopaminergic agents
N04BA	Dopa and dopa derivatives
N04BB	Adamantane derivatives
N04BC	Dopamine agonists
N04BD	Monoamine oxidase B inhibitors
N04BX	Other dopaminergic agents
N03A	Antiepileptics
N03AA	Barbiturates and derivatives
N03AB	Hydantoin derivatives
N03AC	Oxazolidine derivatives
N03AD	Succinimide derivatives
N03AE	Benzodiazepine derivatives
N03AF	Carboxamide derivatives

N03AG	Fatty acid derivatives
N03AX	Other antiepileptics
R03BA	Glucocorticoids
R03BA01	Beclometasone
R03BA02	Budesonide
R03BA03	Flunisolide
R03BA04	Betamethasone
R03BA05	Fluticasone
R03BA06	Triamcinolone
R03BA07	Mometasone
R03BA08	Ciclesonide
N05BB	Diphenylmethane derivatives (sedating)
R03A	Adrenergics, inhalants
R03AC	Selective beta-2-adrenoreceptor agonists
R03AK	Adrenergics and other drugs for obstructive airway diseases
R03C	Adrenergics for systemic use
R03CC	Selective beta-2-adrenoreceptor agonists
R03B	Other drugs for obstructive airway diseases, inhalants
R03BB	Anticholinergics
C01B	Antiarrhythmics, class I and III
C01BA	Antiarrhythmics, class Ia
C01BB	Antiarrhythmics, class Ib
C01BC	Antiarrhythmics, class Ic
C01BD	Antiarrhythmics, class III
C09	Agents acting on the renin-angiotensin system
C09A	ACE inhibitors, plain
C09AA	ACE inhibitors, plain
C09B	ACE inhibitors, combinations
C09BA	ACE inhibitors and diuretics
C09BB	ACE inhibitors and calcium channel blockers
C09	Agents acting on the renin-angiotensin system
C09C	Angiotensin II antagonists, plain
C09CA	Angiotensin II antagonists, plain
C09D	Angiotensin II antagonists, combinations
C09DA	Angiotensin II antagonists and diuretics
C09DB	Angiotensin II antagonists and calcium channel blockers
C09DX	Angiotensin II antagonists, other combinations
C07A	Beta blocking agents
C07AA	Beta blocking agents, non-selective
C07AB	Beta blocking agents, selective
C07AG	Alpha and beta blocking agents
C07B	Beta blocking agents and thiazides
C07BA	Beta blocking agents, non-selective, and thiazides
C07BB	Beta blocking agents, selective, and thiazides
C07BG	Alpha and beta blocking agents and thiazides
C07C	Beta blocking agents and other diuretics
C07CA	Beta blocking agents, non-selective, and other diuretics
C07CB	Beta blocking agents, selective, and other diuretics
C07CG	Alpha and beta blocking agents and other diuretics
C07D	Beta blocking agents, thiazides and other diuretics

C07DA	Beta blocking agents, non-selective, thiazides and other diuretics
C07DB	Beta blocking agents, selective, thiazides and other diuretics
C07F	Beta blocking agents and other antihypertensives
C07FA	Beta blocking agents, non-selective, and other antihypertensives
C07FB	Beta blocking agents, selective, and other antihypertensives
C08	Agents acting on the renin-angiotensin system
C08C	Selective calcium channel blockers with mainly vascular effects
C08CA	Dihydropyridine derivatives
C08CX	Other selective calcium channel blockers with mainly vascular effects
C08D	Selective calcium channel blockers with direct cardiac effects
C08DA	Phenylalkylamine derivatives
C08DB	Benzothiazepine derivatives
C08E	Non-selective calcium channel blockers
C08EA	Phenylalkylamine derivatives
C08EX	Other non-selective calcium channel blockers
C08G	Calcium channel blockers and diuretics
C08GA	Calcium channel blockers and diuretics
C02A	Antiadrenergic agents, centrally acting
C02AA	Rauwolfia alkaloids
C02AB	Methyldopa
C02AC	Imidazoline receptor agonists
C02C	Antiadrenergic agents, peripherally acting
C02CA	Alpha-adrenoreceptor antagonists
C02CC	Guanidine derivatives
C02D	Arteriolar smooth muscle, agents acting on
C02DA	Thiazide derivatives
C02DB	Hydrazinophthalazine derivatives
C02DC	Pyrimidine derivatives
C02DD	Nitroferricyanide derivatives
C02DG	Guanidine derivatives
C02K	Other non-selective calcium channel blockers
C02KA	Alkaloids, excluding rauwolfia
C02KB	Tyrosine hydroxylase inhibitors
C02KC	MAO inhibitors
C02KD	Serotonin antagonists
C02KX	Other antihypertensives
C02L	Calcium channel blockers and diuretics
C02LA	Rauwolfia alkaloids and diuretics in combination
C02LB	Methyldopa and diuretics in combination
C02LC	Imidazoline receptor agonists in combination with diuretics
C02LE	Alpha-adrenoreceptor antagonists and diuretics
C02LF	Guanidine derivatives and diuretics
C02LG	Hydrazinophthalazine derivatives and diuretics
C02LK	Alkaloids, excluding rauwolfia, in combination with diuretics
C02LL	MAO inhibitors and diuretics
C02LN	Serotonin antagonists and diuretics
C02LX	Other antihypertensives and diuretics
C03A	Low-ceiling diuretics, thiazides
C03AA	Thiazides, plain
C03AB	Thiazides and potassium in combination

C03AH	Thiazides, combinations with psycholeptics and/or analgesics
C03AX	Thiazides, combinations with other drugs
C03B	Low-ceiling diuretics, excluding thiazides
C03BA	Sulfonamides, plain
C03BB	Sulfonamides and potassium in combination
C03BC	Mercurial diuretics
C03BD	Xanthine derivatives
C03BK	Sulfonamides, combinations with other drugs
C03BX	Other low-ceiling diuretics
C03C	High-ceiling diuretics
C03CA	Sulfonamides, plain
C03CB	Sulfonamides and potassium in combination
C03CC	Aryloxyacetic acid derivatives
C03CD	Pyrazolone derivatives
C03CX	Other high-ceiling diuretics
C03D	Potassium-sparing agents
C03DA	Aldosterone antagonists
C03DB	Other potassium-sparing agents
C03E	Diuretics and potassium-sparing agents in combination
C03EA	Low-ceiling diuretics and potassium-sparing agents
C03EB	High-ceiling diuretics and potassium-sparing agents
C03X	Other diuretics
C03XA	Vasopressin antagonists
G03C	Estrogens
G03CA	Natural and semi synthetic estrogens, plain
G03CX	Other estrogens
G03D	Progestogens
G03DA	Pregnen-(4) derivatives
G03DC	Estren derivatives
G03F	Progestogens and estrogens in combination
G03FA	Progestogens and estrogens, fixed combinations
G03FB	Progestogens and estrogens, sequential preparations
H03A	Thyroid preparations
H03AA	Thyroid hormones
H03B	Antithyroid preparations
H03BA	Thiouracils
H03BB	Sulphur-containing imidazole derivatives
H03BC	Perchlorates
H03BX	Other antithyroid preparations
M01CB03	Auranofin
M01CB02	Sodium aurothiomalate
M01CC01	Penicillamine
P01BA01	Chloroquine
P01BA02	Hydroxychloroquine sulphate
L04AX01	Azathioprine
L04AD01	Cyclosporine
L04AA13	Leflunomide
L01BA01/L01AX03	Methotrexate
L04AA24	Abatacept
L04AB04	Adalimumab

L04AC03	Anakinra
L04AB01	Etanercept
L04AB02	Infliximab
L01XC02	Rituximab
A07EC01	Sulfasalazine
A10BG	Thiazolidinediones
A10A	Insulins and analogues
A10AB	Insulins and analogues for injection, fast-acting
A10AC	Insulins and analogues for injection, intermediate-acting
A10AD	Insulins and analogues for injection, interm-acting combined with fast-acting
A10AE	Insulins and analogues for injection, long-acting
A10AF	Insulins and analogues for inhalation
A10B	Blood glucose lowering drugs, excluding insulins
A10BA	Biguanides
A10BB	Sulfonamides, urea derivatives
A10BC	Sulfonamides (heterocyclic)
A10BD	Combinations of oral blood glucose lowering drugs
A10BF	Alpha glucosidase inhibitors
A10BH	Dipeptidyl peptidase 4 (DPP-4) inhibitors
A10BX	Other blood glucose lowering drugs, excluding insulins
A10X	Other drugs used in diabetes
A10XA	Aldose reductase inhibitors
A03F	Propulsives
A03FA	Propulsives
A03FA01	Metoclopramide
B01AA	Vit K antagonist
B01AB	Heparin group
N02A	Opioids
N02AA	Natural opium alkaloids
N02AB	Phenylpiperidine derivatives
N02AC	Diphenylpropylamine derivatives
N02AD	Benzomorphan derivatives
N02AE	Oripavine derivatives
N02AF	Morphinan derivatives
N02AG	Opioids in combination with antispasmodics
N02AX	Other opioids
M01AA	Butylpyrazolidines
M01AB	Acetic acid derivatives and related substances
M01AC	Oxicams
M01AE	Propionic acid derivatives
M01AG	Fenamates
M01AH	Coxibs
M01AX	Other antiinflammatory and antirheumatic agents, non-steroids
C10AA01	simvastatin
C10AA02	lovastatin
C10AA03	pravastatin
C10AA04	fluvastatin
C10AA05	atorvastatin
C10AA06	cerivastatin

C10AA07	rosuvastatin
C10AA08	pitavastatin
A02BC01	omeprazole
A02BC02	pantoprazole
A02BC03	lansoprazole
A02BC04	rabeprazole
A02BC05	esomeprazole
L02BG	Enzyme inhibitors
L02BG01	aminoglutethimide
L02BG02	formestane
L02BG03	anastrozole
L02BG04	letrozole
L02BG05	vorozole
L02BG06	exemestane

Table S4 online- Co-morbidity and co-medication at case and control moments in BIFAP and CPRD. Case-crossover study

	BIFAP				CPRD			
	Cases date N=5,412		Crude OR	95% CI	Cases date N=12,853		Crude OR	95% CI
	n	%			n	%		
**Co-morbidities (anytime before case moment)**								
Previous fractures ((including hip/femur and any other)	1010	18.66	NA	NA	2149	16.72	NA	NA
Rheumatoid arthritis (not including osteoporosis)	80	1.48	NA	NA	239	1.86	NA	NA
Osteoporosis	844	15.59	NA	NA	1101	8.57	NA	NA
Paget's disease	24	0.44	NA	NA	34	0.26	NA	NA
Anaemia	879	16.24	NA	NA	1058	8.23	NA	NA
Epilepsy/Seizures	94	1.74	NA	NA	301	2.34	NA	NA
Syncope	665	12.29	NA	NA	530	4.12	NA	NA
Ischaemic heart disease	661	12.21	NA	NA	1142	8.89	NA	NA
Cerebrovascular disease	617	11.40	NA	NA	1072	8.34	NA	NA
Malignant neoplasms	731	13.51	NA	NA	1369	10.65	NA	NA
Inflammatory bowel disease	30	0.55	NA	NA	106	0.82	NA	NA
Obstructive airway disease	423	7.82	NA	NA	921	7.17	NA	NA
Liver disease	136	2.51	NA	NA	156	1.21	NA	NA
Chronic renal failure	254	4.69	NA	NA	131	1.02	NA	NA
Mental disorders (without depression)	139	2.57	NA	NA	334	2.6	NA	NA
Dementia and/or Alzheimers	566	10.46	NA	NA	895	6.96	NA	NA

	BIFAP						CPRD					
**Co-medication**	**N**	**%**	**N**	**%**			**N**	**%**	**N**	**%**		
	**Cases date N=5,412**		**∑controls* (date1-4) N=20,029**		**Crude OR**	**95% CI**	**Cases date N=12,853**		**∑control* (date1-4) N=48,382**		**Crude OR**	**95% CI**
**Glucocorticoids >3months**	97	1.79	304	1.52	1.72	1.17-2.54	173	1.35	564	1.17	173	1.35
**Glucocorticoids (inhaled)**	113	2.09	467	2.33	0.67	0,46-0,96	293	2.28	1220	2.52	293	2.28
**Bisphosphonate use**	304	5.62	1092	5.45	1.26	0,97-1,65	459	3.57	1430	2.96	459	3.57
**Raloxifene**	22	0.41	81	0.40	1.11	0,41-3,01	10	0.08	37	0.08	10	0.08
**Strontium danelate**	11	0.20	56	0.28	0.48	0,15-1,49	20	0.16	46	0.10	20	0.16
**Parathyroid hormone**	9	0.17	16	0.08	14.24	1,65-122,94	0	0.00	0	0.00	0	0.00
**Calcium & Vitamin D**	451	8.33	1638	8.18	1.12	0,93-1,34	765	5.95	2301	4.76	765	5.95
**Calcitonin**	54	1.00	172	0.86	1.39	0,89-2,16	2	0.02	8	0.02	2	0.02
**Antidepressants**	1332	24.61	4511	22.52	1.72	1,49-1,98	1580	12.29	5318	10.99	1580	12.29
**Antipsychotics/lithium**	481	8.89	1511	7.54	1.54	1,30-1,83	901	7.01	2924	6.04	901	7.01
**Anti-Parkinsons drugs**	262	4.84	911	4.55	1.97	1,28-3,02	268	2.09	930	1.92	268	2.09
**Anticonvulsants**	463	8.56	1562	7.80	1.52	1,23-1,88	474	3.69	1584	3.27	474	3.69
**Bronchodilators**	526	9.72	1915	9.56	1.11	0,92-1,34	782	6.08	2972	6.14	782	6.08
**Antihypertensives**	2615	40.00	8203	40.96	0.95	0,84-1,09	2334	18.16	9055	18.72	2334	18.16
**Diuretics**	1385	25.59	5110	25.51	1.11	0,97-1,27	1990	15.48	7573	15.65	1990	15.48
**Anti-arrhythmics**	131	2.42	421	2.10	1.90	1,17-3,09	146	1.14	533	1.10	146	1.14

**Sedating antihistamines**	67	1.24	229	1.14	1.22	0,84-1,78	33	0.26	108	0.22	33	0.26
**Estrogen-containing hormone replacement therapy (HRT)**	10	0.18	36	0.18	1.16	0,38-3,50	75	0.58	318	0.66	75	0.58
**Thyroid hormones**	205	3.79	726	3.62	1.53	1,03-2,26	597	4.64	2229	4.61	597	4.64
**Antithyroid drugs**	23	0.42	86	0.43	1.07	0,44-2,62	20	0.16	61	0.13	20	0.16
**Disease-modifying anti-rheumatic drugs (DMARDs)**	42	0.78	177	0.88	0.80	0,42-1,50	107	0.83	406	0.84	107	0.83
**Thiazolidinediones**	22	0.41	73	0.36	2.02	0,76-5,38	37	0.29	137	0.28	37	0.29
**Other antidiabetics**	718	13.27	2842	14.19	0.68	0,54-0,85	423	3.29	1587	3.28	423	3.29
**Antiemetic (Metoclopramide)**	83	1.53	260	1.30	1.24	0,93-1,64	117	0.91	364	0.75	117	0.91
**Anticoagulants**	509	9.41	1574	7.86	1.96	1,62-2,37	309	2.40	1168	2.41	309	2.40
**Morphine/opiates**	613	11.33	1860	9.29	1.88	1,61-2,20	1373	10.68	4424	9.14	1373	10.68
**=>2 prescriptions for a non-steroidal anti-inflammatory drug (NSAID)**	514	9.50	2079	10.38	0.85	0,74-0,98	440	3.42	1634	3.38	440	3.42
**Statins**	680	12.56	2675	13.36	0.87	0,70-1,06	1199	9.33	4548	9.40	1199	9.33
**Proton pump inhibitors**	2015	37.23	6920	34.55	1.56	1,40-1,74	1681	13.08	5863	12.12	1681	13.08
**Aromatase inhibitors**	34	0.63	141	0.70	0.77	0,35-1,69	64	0.50	223	0.46	64	0.50

*Sum of controls columns report number of control moments exposed to each co-morbidity.
NA: Non-applied due to the matching by patient.
